# Availability and Uptake of Cervical Cancer Screening and Treatment Services at 19 Kenyan Health Facilities: A Brief Report

**DOI:** 10.1200/GO-24-00255

**Published:** 2025-04-11

**Authors:** Catherine Wexler, May Maloba, Natabhona Mabachi, Nicodemus Maosa, Shadrack Babu, Vincent S. Staggs, Sharon Mokua, Kathy Goggin, Sarah Finocchario-Kessler

**Affiliations:** 1 Department of Family Medicine and Community Health, University of Kansas Medical Center, Kansas City, KS; 2 Global Health Innovations—Kenya, Nairobi, Kenya; 3 The DARTNet Institute, Aurora, CO; 4 Health Services and Outcomes Research, Children’s Mercy Kansas City, Kansas City, MO; 5 Kenya Medical Research Institute, Nairobi, Kenya; 6 Schools of Medicine and Pharmacy, University of Missouri—Kansas City, Kansas City, MO

## Abstract

**PURPOSE:**

In 2012, Kenya announced its commitment to prioritizing cervical cancer (CC) services with the National Cervical Cancer Prevention Program. However, gaps in implementation remain, causing suboptimal uptake. The goal of this study is to provide a situational analysis of 19 government health facilities in Kenya to assess facilities’ capacity to provide CC screening and treatment.

**METHODS:**

We conducted a retrospective data review at 19 health facilities in Siaya and Busia counties of Kenya from September 2021 to September 2022 to assess availability and uptake of CC services. Key variables included hospital volume, positivity, and numbers of women screened, treated, and referred. Notes were taken to document challenges with service provision and data collection. The retrospective review informed site matching for a proposed cluster-randomized trial.

**RESULTS:**

There were a total of 16,757 CC screenings or rescreenings documented across the 19 study sites during the study period. Across the 19 facilities, 10.8% of the women enrolled in care at the hospital were screened during the data collection period. The overall positivity was 3.8% but ranged from 0.7% to 16.7%. Of the 19 facilities offering CC screening, five lacked the required equipment to provide same-day treatment, and complex referral pathways were noted for women needing to seek care elsewhere. Challenges with documentation were noted at all stages of CC services.

**CONCLUSION:**

Gaps noted were low screening rates, opportunities to improve the efficiency of CC services, structural limitations in implementing the screen-and-treat approach, and poor documentation of CC services and outcomes. Urgent action is needed to ensure that all facilities offering CC screening are equipped with the personnel, supplies, and equipment necessary to conduct guidelineadherent same-day cryotherapy or thermal ablation treatment and improve documentation at the facility level to track CC services across the cascade of care.

## INTRODUCTION

Cervical cancer (CC) is preventable; yet in 2020, an estimated 604,000 cases were diagnosed and 342,000 deaths occurred globally.^1^ Over a third of these deaths occurred in sub-Saharan Africa although only 14% of the world’s female population lives in the region.^2^ In Kenya, around 5,250 new cases ofCC are diagnosed annually and over 3,200 women die of CC every year.^3^ To achieve population-level health gains, the WHO recommends countries reach 70% coverage with screening and 90% treatment coverage.^4^

In 2012, Kenya launched the National Cervical Cancer Prevention Program which outlined its commitment to reduce the burden of CC.^5^ These priorities include scaling up the availability of CC prevention services at all public hospitals, enhancing referral networks between facilities, and integrating CC screening and treatment indicators into the health management information systems. While policy has continued to evolve and improve since then, implementation of these policies remains suboptimal. Current guidelines call for a single-visit screen-and-treat approach, in which a woman is screened by visual inspection with acetic acid (VIA) or visual inspection with Lugol’s iodine (VILI) and precancerous lesions receive same-day treatment using thermal ablation or cryotherapy.^6^ VIA/VILI can be conducted at lower-level health facilities; however, women found to have advanced lesions need to be treated at a higher-level facility for additional diagnostics and management. VIA/VILI screening and treatment of precancerous lesions through cryotherapy are free of charge for Kenyan women.^7^ However, additional diagnostics (eg, biopsies) are more expensive, averaging $181.38 in US dollars (USD), and advanced treatments (chemotherapy, radiation) range from $1,575.93 to $6514 (USD),^8,9^ whereas health insurance coverage to help offset these costs remains below 20% of the population.^10^

In Kenya, patient- and system-level barriers contribute to low uptake ofCC screening and treatment. Estimates suggest that only 10%-36% of women age 18-69 years have received effective CC screening,^11–16^ and estimates suggest that only 22%-39% of women diagnosed with precancerous lesions receive treatment.^16,17^ At the system level, lack of trained personnel, insufficient space, and supply shortages pose barriers to guideline-adherent screening.^16,18,19^ Furthermore, high costs of treatment, limited availability of centers equipped to treat provide treatment, and lack of trained providers pose barriers to women who have a positive screen.^16,20^ At the patient level, inadequate knowledge, discomfort/embarrassment over the procedure, wait time, and lack of financial resources to pay for treatment challenge the uptake of screening and treatment.^20,21^

The goal of this study is to provide a situational analysis of19 government health facilities in a region of Kenya with high HIV prevalence to assess facilities’ capacity to provide CC screening and treatment, key indicators ofCC screening and treatment, and challenges to providing guideline-adherent care. These data were collected to inform matching of 10 sites for a proposal for a cluster randomized controlled trial to evaluate an intervention to improve CC services.

## METHODS

We conducted a retrospective data review at 19 health facilities in Siaya and Busia counties. Facilities were included if they (1) were government-run, (2) were designated as a health center, subcounty, county, or county referral hospital, and (3) offered CC screening services. We trained 10 research assistants (RAs) to conduct retrospective record review. Data collection tools were developed for the purpose of the study. Before study launch, the tools were pilot tested at one facility and all RAs received training on study objectives, ethical considerations, uniformity of data entry, trouble-shooting missing data points, and qualitative interviewing and documentation. Permission for retrospective data collection was obtained from each facility. The study was approved by the Institutional Review Board at the University of Kansas Medical Center (STUDY00149289).

Data collection occurred in November 2022. Both data on facility indicators and aggregate patient indicators were collected. For facility indicators, RAs noted the availability of different screening modalities, the availability of different treatment modalities, the number of providers trained on each screening and treatment modality, and referral pathways when screening and treatment could not be conducted on site. These facility indicators were assessed through discussions with providers and administration and visual observations of presence and functionality of equipment.

For patient indicators, RAs reviewed CC registries, electronic medical records, patient files, and monthly reports to document the aggregate number of clients receiving services from September 2021 to September 2022. Key data points collected included outcomes related to the number of CC screenings conducted, the number of abnormal CC results, the number of clients with documented follow-up (cryotherapy, thermal ablation, Loop Electrosurgical Excision Procedure [LEEP], other treatment, referral to other facilities) after abnormal screen, and the number of patients who were referred, and received treatment, elsewhere. Quantitative data were analyzed using descriptive statistics and counts. Field notes from informal discussions with providers about CC service provision challenges were documented. All data were entered into an online database (RedCap). RAs were encouraged to note challenges in the corresponding section of the data collection tool to help contextualize findings. Notes from providers and RA-documented challenges were summarized by the lead author and described.

## RESULTS

### Facility Indicators

Facility levels and sizes are described in Table 1.

All 19 health facilities offered screening through VIA/VILI. The number of providers trained to provide VIA/VILI ranged from 2 to 10. The ratio of the number of VIA/VILI tests conducted per year to the number of providers trained on VIA/VILI ranged from 18:1 (at a health center) to 1,083:1 (at a referral hospital). Five facilities offered Pap smear tests, and four offered human papillomavirus (HPV) testing.

In terms of treatment availability, 14 facilities had a thermal ablation and/or cryotherapy capability, with the other five (26%, Hospital IDs 8, 9, 13, 18, 19) having neither. Only two facilities offered LEEP, one offered chemotherapy, and none offered radiation therapy. There was wide variability across facilities in the number of providers trained for VIA/VILI (2–10), cryotherapy (0–6), and thermal ablation (1–6). No facility had more than one provider trained in LEEP.

### Patient Outcomes

The total number of CC screenings documented across the 19 study sites during the study period was 16,757, including 11,691 initial screenings and 5,066 rescreenings. The number of CC screenings conducted at facilities in a year ranged from 54 to 4,400, which represented between 1.2% and 49.1% of the total number of women enrolled in care at the hospital. The percent of abnormal screens ranged from 0.4% to 16.7%. Among women with abnormal screening results, the percentage with documented follow-up varied drastically from 0% to 100%. Across all facilities, 10.8% of women enrolled received screening, and among the 3.8% with an abnormal result, 20.1% had no documented follow-up (Table 2).

### Challenges to CC Screening and Treatment

Through informal discussions with providers and field notes, RAs documented several challenges that affect the provision of guideline-adherent CC care, most notably inconsistent documentation, lack of coordination between multiple screening points, complex referral pathways, and inadequate hospital resources.

Inconsistent documentation across the CC cascade of care was a major challenge. Starting from the point of screening, it was difficult for RAs to determine who was eligible for screening based on the existing data collection tools. This was exacerbated by a lack of coordination between multiple screening points within the same facility. Screening points maintained their own registers and documentation that were not consistently shared across departments; thus, no single, comprehensive record of CC services was maintained at the facility level.

Complex and disorganized referral pathways created inefficiencies and barriers. Women seeking care in facilities without on-site treatment would need to move between multiple facilities to receive complete care. For example, one facility had neither thermal ablation nor cryotherapy available and would refer patients to a second facility for care. This facility, however, only had thermal ablation available, so more advanced patients would be referred to a third facility. In cases of invasive CC requiring advanced treatments, patients would then be referred to a fourth facility at the county or national level. Thus, a woman with invasive CC might have to move between 3 and 4 facilities before ending up at a facility equipped to provide her complete guideline-adherent care. Documentation of patients’ postreferral follow-up was poor, so we were unable to include the percentage of referred patients who received complete guideline-adherent services as an outcome in any meaningful way.

RAs also noted intermittent lack of basic commodities (eg, gloves, disposable speculums, screening reagents, and gas for cryotherapy) for CC screening and treatment. Staffing shortage and inconsistent power supply at the hospitals were also noted as resource limitations that affected service delivery.

## DISCUSSION

In 2020, the WHO released its 90-70-90 goals for CC prevention. Goals include (1) 90% of girls being fully vaccinated with HPV vaccine by age 15 years, (2) 70% of women to receive CC screening at least two times between age 30 and 45 years, and (3) 90% of women identified with cervical disease to receive treatment. Kenya has strengthened its CC screening and treatment services over the past decade; however, gaps in the implementation of these best practices hinder the achievement of global and national goals for CC screening and treatment. The major gaps we noted in this study include low screening rates, opportunities to improve the efficiency of CC services, structural limitations in implementing the screen-and-treat approach, and poor documentation of CC services and outcomes. Similar gaps were noted by the National Cervical Cancer Stakeholders Consultative Forum.^17^

Consistent with others,^16,22^ our study indicates that screening rates are well below the target. We observed that between 1% and 49% of all women seeking care in a facility were screened during the previous year, with an average of 10.8% across all facilities. At the facility level, there are no mechanisms in place to assess the number of women eligible for CC screening, and thus, estimates do not account for women outside of the screening target parameters or those ineligible for screening based on past screening. However, nationally representative modeling indicates that <40% of eligible women without HIV and <10% of eligible women living with HIV were screened annually.^16^ Furthermore, the inability to assess screening rates among eligible women at the facility level is a major limitation to assessing progress in CC programs.

The globally accepted benchmark for VIA positivity is 5%-10%, with even higher positivity (25%1) expected among populations with high HIV prevalence or screening-naïve populations.^23^ The WHO notes that a VIA positivity rate of <3% indicates a lack of quality care and should trigger corrective action.^23^ In our study, the combined positivity across 19 health facilities was 3.8%, with seven of 19 health facilities averaging 3% positivity or less. Additional mentorship and training may be required to ensure that providers are able to correctly identify abnormal VIA/VILI results. While other screening methods, such as HPV testing, have higher sensitivity and less subjectivity than VIA/VILI, challenges of infrastructure, cost, and test processing time precluding same visit treatment limit their use in low resource settings. Point-of-care HPV tests, such as GeneXpert, may overcome these challenges; however, they are not widely available in Kenya.^24^

While same-visit screen-and-treat approach is the recommended method, 26% of facilities in our survey did not have the capability to provide same-day treatment of precancerous lesions. Instead, these facilities referred patients to other facilities, without appropriate mechanisms of tracking them introducing opportunities for delayed care and loss to follow-up. Poor documentation of CC follow-up was noted across facilities and challenged our ability to get comprehensive data on targeted outcomes. This lack of coordinated data collection in health care is not unique to Kenya, with a multinational study indicating that nearly 50% of CC records across eight countries in sub-Saharan Africa had no documented follow-up or were unable to be traced.^25^ The same study found that Kenya had the highest rate of guideline-adherent CC care at 49%; however, this study was conducted in Eldoret and Nairobi, major cities with locally available oncology centers.

Poor documentation and uncoordinated departments and referrals challenged data collection. While we used multiple records and registries, some targeted data were not available, and there were large gaps in the available data. This lack of documentation is, in its own way, a study finding and highlights the critical importance of improving documentation to more accurately assess progress made toward achieving global CC screening and treatment goals. Given our larger objective of collecting data for site matching for an upcoming CC study, only facilities that had active CC screening programs were included. In 2018, only 25% of nearly 3,000 facilities surveyed offered CC screening^16^; thus, this study is not representative of up to 75% of facilities that do not offer screening. Finally, as we only collected aggregate data, we do not have any information on sociodemographic characteristics of the population that these data are drawn from. We were also unable to disaggregate screening rates among women living with HIV and those without.

Nevertheless, this study provides important insight into the availability of CC screening and treatment services in Kenya. Urgent action is needed to improve documentation at the facility level to track CC screening and treatment services across departments within a single facility and referrals across facilities. Investment from the county, national, and donor levels is needed to ensure that all facilities offering CC screening are equipped with the personnel, supplies, and equipment necessary to conduct guideline-adherent same-day cryotherapy or thermal-ablation treatment. Finally, training is needed to ensure that providers conducting VIA/VILI are accurately diagnosing patients.^26^

## Figures and Tables

**TABLE 1.** Characteristics of the 19 Included Facilities

Hospital Identifier	Hospital Level	County	Women Enrolled in Care During the Study Period
1	Referral	Busia	51,551
2	Subcounty	Siaya	21,368
3	Subcounty	Busia	8,500
4	County	Siaya	8,219
5	County	Siaya	7,196
6	Health center	Siaya	7,060
7	Subcounty	Siaya	6,236
8	Subcounty	Busia	6,000
9	Subcounty	Busia	5,450
10	Subcounty	Busia	5,065
11	Subcounty	Busia	4,975
12	District	Siaya	4,780
13	Health center	Siaya	4,562
14	Subcounty	Busia	4,156
15	Subcounty	Busia	4,141
16	Health center	Siaya	2,789
17	Health center	Busia	1,531
18	Health center	Siaya	934
19	District	Siaya	640

**TABLE 2.** Cervical Cancer Service Outcomes, by Facility and Combined

Hospital Identifier	No. of Women Enrolled	No. of Screenings/Rescreenings	Women Screened (%)	No. of Abnormal Screens	Abnormal (%)	Abnormal With No Follow-Up	Abnormal With No Follow-Up (%)
1	51,551	4,440	8.6	160	3.6	18	11.3
2	21,368	1,878	8.8	13	0.7	8	61.5
3	8,500	1,087	12.8	77	7.1	16	20.8
4	8,219	2,712	33.0	104	3.8	0	0.0
5	7,196	477	6.6	8	1.7	0	0.0
6	7,060	1,003	14.2	4	0.4	0	0.0
7	6,236	617	9.9	11	1.8	6	54.5
8	6,000	478	8.0	33	6.9	8	24.2
9	5,450	359	6.6	12	3.3	7	58.3
10	5,065	730	14.4	24	3.3	6	25.0
11	4,975	490	9.9	17	3.5	5	29.4
12	4,780	272	5.7	36	13.2	24	66.7
13	4,562	54	1.2	3	5.6	3	100.0
14	4,156	615	14.8	22	3.6	4	18.2
15	4,141	544	13.1	91	16.7	15	16.5
16	2,789	260	9.3	2	0.8	0	0.0
17	1,531	301	19.7	12	4.0	0	0.0
18	934	126	13.5	2	1.6	2	100.0
19	640	314	49.1	6	1.9	6	100.0
Combined	155,153	16,757	10.8	637	3.8	128	20.1

## Data Availability

The data sets used and/or analyzed during the current study are available from the corresponding author on reasonable request.

## CONTEXT

### Key Objective

To assess cervical cancer (CC) screening and treatment capacity and uptake at 19 facilities in Kenya.

### Knowledge Generated

All 19 health facilities offered screening through visual inspection with acetic acid/visual inspection with Lugol’s iodine, but five (26%) did not have any treatment capacity, precluding their ability to offer a guideline-adherent screen-and-treat approach. Only 10.8% of women enrolled at the 19 facilities received screening during the study period, with a positivity rate of 3.8% and 20.1% of women with a positive screen having no documented follow-up.

### Relevance

Investment from the county, national, and donor levels is needed to ensure that all facilities offering CC screening are equipped with the personnel, supplies, and equipment necessary to conduct guideline-adherent same-day cryotherapy or thermal ablation treatment.
